# Diagnosis for autism spectrum disorder children using T1-based gray matter and arterial spin labeling-based cerebral blood flow network metrics

**DOI:** 10.3389/fnins.2024.1356241

**Published:** 2024-04-17

**Authors:** Mingyang Liu, Weibo Yu, Dandan Xu, Miaoyan Wang, Bo Peng, Haoxiang Jiang, Yakang Dai

**Affiliations:** ^1^School of Electrical and Electronic Engineering, Changchun University of Technology, Changchun, China; ^2^Department of Radiology, Affiliated Children’s Hospital of Jiangnan University, Wuxi, China; ^3^Suzhou Institute of Biomedical Engineering and Technology, Chinese Academy of Science, Suzhou, China

**Keywords:** autism spectrum disorder, T1-weighted MRI, ASL, gray matter network, cerebral blood flow network, machine learning

## Abstract

**Introduction:**

Autism Spectrum Disorder (ASD) is a complex neurodevelopmental condition characterized by impairments in motor skills, communication, emotional expression, and social interaction. Accurate diagnosis of ASD remains challenging due to the reliance on subjective behavioral observations and assessment scales, lacking objective diagnostic indicators.

**Methods:**

In this study, we introduced a novel approach for diagnosing ASD, leveraging T1-based gray matter and ASL-based cerebral blood flow network metrics. Thirty preschool-aged patients with ASD and twenty-two typically developing (TD) individuals were enrolled. Brain network features, including gray matter and cerebral blood flow metrics, were extracted from both T1-weighted magnetic resonance imaging (MRI) and ASL images. Feature selection was performed using statistical *t*-tests and Minimum Redundancy Maximum Relevance (mRMR). A machine learning model based on random vector functional link network was constructed for diagnosis.

**Results:**

The proposed approach demonstrated a classification accuracy of 84.91% in distinguishing ASD from TD. Key discriminating network features were identified in the inferior frontal gyrus and superior occipital gyrus, regions critical for social and executive functions in ASD patients.

**Discussion:**

Our study presents an objective and effective approach to the clinical diagnosis of ASD, overcoming the limitations of subjective behavioral observations. The identified brain network features provide insights into the neurobiological mechanisms underlying ASD, potentially leading to more targeted interventions.

## 1 Introduction

Autism spectrum disorder (ASD) is a neurodevelopmental condition marked by disruptions in social, emotional, and cognitive brain connectivity, significantly affecting an individual’s daily functioning. However, diagnosing ASD presents formidable challenges. While typically diagnosed between the ages of 3 and 6, symptoms often manifest as early as 1 to 3 years of age. However, objectively identifying these symptoms is a complex task (Maenner et al., 2021; Yuan and Luo, 2022). Currently, assessments primarily rely on clinical symptoms and scales behavioral observations and subjective judgments. The assessment of clinical symptoms and scales typically involves subjective judgments. Different doctors or assessors may arrive at different conclusions. Meanwhile, clinical symptom assessments and scales primarily focus on observable behaviors while overlooking the structural and functional variances within the patient’s brain. Magnetic resonance imaging provides objective information about brain structure and function, enabling doctors to make diagnoses without relying on subjective observations. This helps reduce the subjectivity of ASD diagnoses (Klin et al., 2000; Koumpouros and Kafazis, 2019).

In previous studies, researchers have employed brain network characteristics based on resting-state functional magnetic resonance imaging (fMRI) to classify individuals with ASD compared to healthy controls (Nebel et al., 2016; Ingalhalikar et al., 2021; Yang et al., 2022). These methods, through functional connectivity analysis, have unveiled developmental abnormalities within the ASD patients, providing insights into the intricacies of intra- and inter-network functional connectivity. When contrasting brain network features between children with ASD and typically developing individuals, it becomes evident that children with ASD exhibit a reduced density of brain network connections, particularly in higher frequency bands (Byeon et al., 2020). This indicates that the transfer of information between brain regions in children with ASD is irregular or constrained, thereby giving rise to specific characteristics in their social and cognitive functions.

In addition to resting-state functional magnetic resonance imaging (fMRI), other MRI techniques can also be used to detect the abnormalities in brain structure and function alteration in ASD. T1-weighted imaging is a commonly used magnetic resonance imaging (MRI) technique that primarily reflects the anatomical structure of tissues and the contrast between tissues such as gray matter and white matter. In ASD research, T1-weighted is often used to observe structural changes in the brain, such as gray matter volume, cortical thickness, and connectivity between brain regions (Jianfeng et al., 2019; Conti et al., 2020). By comparing T1-weighted images of ASD patients with healthy controls, researchers can discover structural differences in certain brain regions of ASD patients, which may be related to cognitive, social, and emotional disorders in ASD (Alvarez-Jimenez et al., 2020; Samian et al., 2021). Arterial spin labeling (ASL) is a non-invasive imaging technique used to study brain function and neural activity. The signal of fMRI mainly comes from the local blood oxygen level-dependent (BOLD) effect caused by neuronal activity. The BOLD signal is only an indirect and qualitative measurement of blood supply, and its hemodynamic response function may be abnormal in autistic individuals, which may affect the reliability and accuracy of the study. In contrast, ASL can directly and objectively measure local and global brain perfusion intensity and blood flow. ASL’s measurement of blood flow is not affected by blood oxygen status, and blood flow is significantly correlated with neural activity (Ohnishi et al., 2000; Lindner et al., 2017). CBF is of significant importance in studying abnormalities in brain perfusion. The measurement of CBF lateralization in temporal lobes was used as a discriminative feature for diagnosis ASD (Wong et al., 2017; Lin et al., 2023).

In addition to abnormalities in specific brain regions, interactions among brain regions in ASD are also atypical. Research indicates that the connections between different brain areas in ASD patients may differ from those in typically developing individuals, potentially affecting the social and cognitive function of ASD patients. These abnormalities may involve multiple brain regions, including those related to sensory perception, motor functions, emotions, and social functions (Khan et al., 2015; Abbott et al., 2016; Guo et al., 2016). Previous studies have used resting-state fMRI to construct functional brain networks and, in combination with the support vector machine recursive feature elimination (SVM-RFE) classification method, classify ASD patients and typically developing controls (Wang et al., 2019). A study used structural MRI data collected from 817 subjects aged 7–64 years sourced from the ABIDE-I database (Rakić et al., 2020). They downscaled the feature vectors using Fisher’s algorithm and employed auto-encoder and multilayer perceptron algorithms for recognition purposes. Furthermore, some researchers have integrated both gray matter brain networks, which primarily focus on brain structure, and functional brain networks, which emphasize brain activity, to examine ASD (Wang et al., 2023). Combining these approaches provides a more comprehensive understanding that encompasses both structural features and functional connections of the brain.

The aim of this study is to propose an innovative approach to diagnosing ASD by utilizing T1-based gray matter and ASL-based cerebral blood flow network metrics. By integrating gray matter networks and cerebral blood flow networks, researchers can gain a more comprehensive understanding of the brain of ASD children, which provide insights into brain structural abnormalities and changes in perfusion. This proposed approach enables an in-depth exploration of the structural characteristics and functional connections (FC) within ASD children. When considering both structure and function, it contributes to improved diagnostic accuracy for ASD. Consequently, the proposed method is conducive to early and accurate diagnosis of autism, facilitating the discovery of neuroimaging biomarkers and the investigation of neurodevelopmental aberrations in ASD.

## 2 Materials and methods

### 2.1 Participants

This study involved 30 preschool-aged patients with ASD and 22 typically developing (TD) individuals. Table 1 records the clinical information, Gesell Developmental Scale, and Autism Behavior Checklist (ABC) scale information for all subjects. All participants signed a written informed consent after a detailed description of the research. This study was approved by the Institutional Review Board of the Children’s Hospital affiliated with Jiangnan University.

**Table 1:** Demographic and clinical characteristics of the 52 subjects.

	ASD	TD	*p*-value
Number	30	22	—
Age	4.71 ± 1.41	5.49 ± 1.68	0.076
Male/female	24/6	14/8	0.0004
DQ	55.42 ± 11.69	NA	—
Gesell-adaptability	60.04 ± 13.48	NA	—
Gesell-great sports	68.46 ± 18.77	NA	—
Gesell-fine motion	58.81 ± 16.28	NA	—
Gesell-language	41.00 ± 14.88	NA	—
Gesell-personal socializing	49.85 ± 12.78	NA	—
ABC-total	69.16 ± 24.42	NA	
ABC-sensory	9.77 ± 5.44	NA	—
ABC-relating	10.76 ± 5.85	NA	—
ABC-body and object use	10.00 ± 8.30	NA	—
ABC-language	16.82 ± 4.30	NA	—
ABC-social and self-help skills	12.88 ± 5.35	NA	—
CARS	36.10 ± 2.40	NA	—

ABC, Autism Behavior Checklist; DQ, developmental quotient; CARS, Childhood Autism Rating Scale.

### 2.2 MRI acquisitions

The T1-weighted MRI images of all subjects were acquired using a Siemens 1.5T system with the following scanning parameters: TR (Repetition Time)/TE (Echo Time) = 2000/3.1 ms, slice thickness = 1 mm, flip angle (FA) =8°, and field of view (FOV) =90.625. The ASL MRI images were obtained using the same scanner with the following parameters: TR/TE = 4600/15.9 ms, slice thickness = 3 mm, FA = 180°, and FOV = 100.

### 2.3 Data preprocessing

In this research, we used a standard procedure to preprocess the MRI of each subject (Peng et al., 2017), and the T1 images were processed as follows: first, the original T1 MRI images were resampled and reoriented so that the image size was uniformly 256 × 256 × 256 and the spatial resolution was uniformly 1 × 1 × 1; then, cranial stripping and removal of non-brain tissue parts not related to the experiment, such as scalp, skull and dura mater and other extra-brain tissues, were performed; then, brain tissue segmentation was performed to separate the brain tissues in the images, and further brain parenchyma segmentation was performed. Then, region of interest (ROI) was labeled using the automatic anatomical labeling (AAL) template (Tzourio-Mazoyer et al., 2002). Finally, cortical surface reconstruction was performed to calculate cortical thickness and area (Wang et al., 2014). This preprocessing pipeline ensured standardization and consistency of the MRI data, facilitating subsequent analysis and interpretation of brain connectivity and structural alterations in children with ASD.

For ASL images, we used the following alignment procedure: first, the CBF image was calculated based on the different time series, and then the CBF image was redirected and resampled to make the different modal images of each subject have the same size and number of layers. Then, FMRIB’s Software Library (FSL) software was used, and the linear transformation FMRIB’s Linear Image Registration Tool (FLIRT) function was employed to use the mutual information function as the cost of the T1 image after skull peeling as the reference image and the CBF image as the floating image. These preprocessed images can then be used for feature extraction and analysis.

### 2.4 Brain network metrics computation

For T1-based gray matter network, we selected 78 cortical regions (excluding subcortical brain regions in AAL template). We then calculated the cortical thickness within each cortical region and obtained a 78 × 78 brain network metric by calculating the Pearson correlation coefficients in Eq. 1 and Eq. 2.

(1) $$f\left(a,b\right)=\exp\left(\frac{{\left[u\left(a\right)-u\left(b\right)\right]}^{2}}{2\partial^{2}}\right)$$

(2) $$\partial =\sqrt{\partial_{a}^{2}+\partial_{b}^{2}}$$

where $u\left(a\right)$ and $u\left(b\right)$ are the cortical thicknesses, $\partial_{a}$ and $\partial_{b}$ are the standard deviations of brain region a and b , ${\left[u\left(a\right)-u\left(b\right)\right]}^{2}$ characterizes the correlation between brain regions, of the cortical thicknesses. We extracted the upper triangle of this matrix to generate a feature vector of T1-based gray matter network for each subject.

For ASL-based cerebral blood flow network, we extracted CBF features of 78 ROI region from ASL images, constructed an ASL brain network using Pearson correlation, and a 78 × 78 ASL-based brain network metrics was obtained. The feature vector was gained as the same approach of T1-based brain network metric. After feature vector extraction, *z*-score normalization was used to change the feature values to a common scale.

### 2.5 Machine learning based diagnosis pipeline

**Figure 1:**
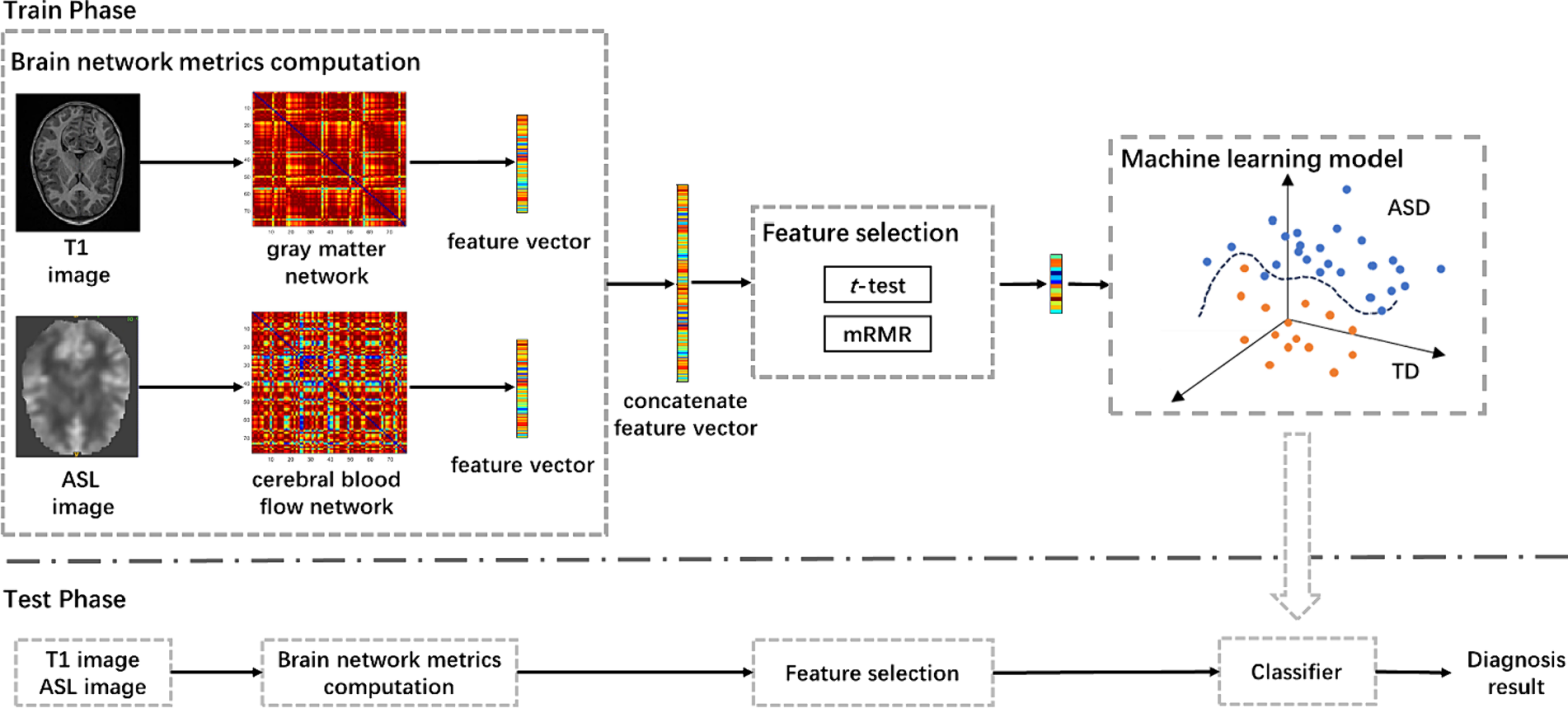
The pipeline of the proposed approach for ASD diagnosis using T1-based gray matter and ASL-based cerebral blood flow network metrics, including brain network metrics computation, feature selection, and machine learning model. mRMR, minimum redundancy and maximum relevance; ASD, autism spectrum disorder.

The pipeline of the proposed approach is shown in Figure 1, including brain network metrics computation, feature selection, and machine learning model. In the feature selection module, the statistical *t*-test and the minimum redundancy maximum relevance (mRMR) were employed to select feature. Initially, the statistical *t*-test was utilized to select features with *p*-value below 0.05. Subsequently, the mRMR method was applied to further reduce the dimensionality of the brain network features (Peng et al., 2005). In the machine learning module, we used a random vector functional link (RVFL) classification framework (Pao et al., 1994).

We compared our ASD classification performance with that using only T1-based gray matter volume and ASL-based cerebral blood flow network metrics as features. Besides, we also adopted several other classification models for performance comparison, including support vector machine (SVM) (Vapnik, 1964) and extreme learning machine (ELM) (Huang et al., 2004).

### 2.6 Statistical analysis

All statistical analyses were conducted using SPSS (v26.0; IBM SPSS Statistics for Windows, NY, Armonk, United States). Gender differences between the ASD patients and TD were examined using chi-square tests. Two-sample *t*-tests were used to assess differences in continuous measures between groups. To assess the performance of the classification model, we used a variety of metrics, including accuracy (ACC), sensitivity (SEN), specificity (SPC), precision, F1-score, area under the receiving operating characteristic curve (AUC). These metrics were used to validate the effectiveness and predictive power of the classification model.

**Figure 2:**
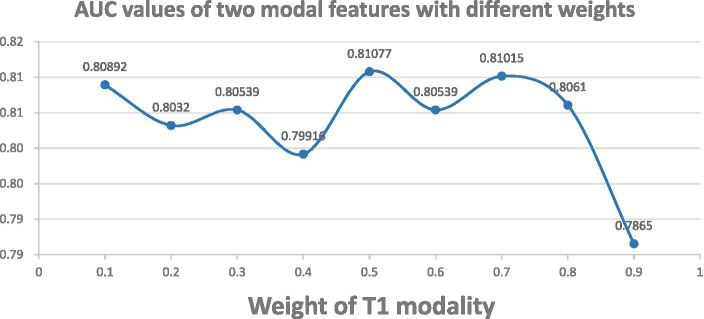
AUC plots for different weight combinations. Demonstrates the change of ASD classification AUC curves when adjusting the weights of T1 gray matter and cerebral blood flow network features. T1 gray matter weights 0.1–0.9, cerebral blood flow weights 0.9–0.1.

To validate the robustness of our method, we adjusted the weights of T1 gray matter network features and cerebral blood flow network features. Specifically, we adjusted the weight of T1 gray matter network features within the range of 0.1 to 0.9, and correspondingly adjusted the weight of cerebral blood flow network features within the range of 0.9 to 0.1. As shown in Figure 2, when the weights of both T1 gray matter network features and cerebral blood flow network features were 0.5, the classification results reached their best state, and the AUC value also reached its highest. This result indicates that both types of features have equal importance in the classification process and can maximize the classification effect when their weights are equal. To further enhance the stability of the model, we adopted a five-fold cross-validation strategy and repeated the experiment 20 times to ensure its good stability across different data divisions.

## 3 Results

### 3.1 The performance of the proposed method in ASD diagnosis

To comprehensively evaluate the performance of our proposed framework, we compared with SVM and ELM classifiers and scientifically assessed the performance differences between them and the RVFL classifier using the DeLong test. The experimental results in Table 2 show that SVM and ELM achieved accuracies of 59.63 and 81.45%, respectively, in ASD diagnosis, while the RVFL classifier achieved the best accuracy of 84.30%.

**Table 2:** Classification results based on different classifiers in ASD recognition.

Classification	ACC	SEN	SPC	F1-score	precision	AUC	*p*-value
SVM	59.63%	50.00%	75.10%	84.34%	80.81%	0.52	<0.001
ELM	81.45%	87.96%	74.83%	60.14%	75.24%	0.47	<0.001
RVFL	84.30%	82.67%	85.56%	81.34%	86.37%	0.81	—

RVFL, random vector functional link; ELM, extreme learning machine; SVM, support vector machine.

**Figure 3:**
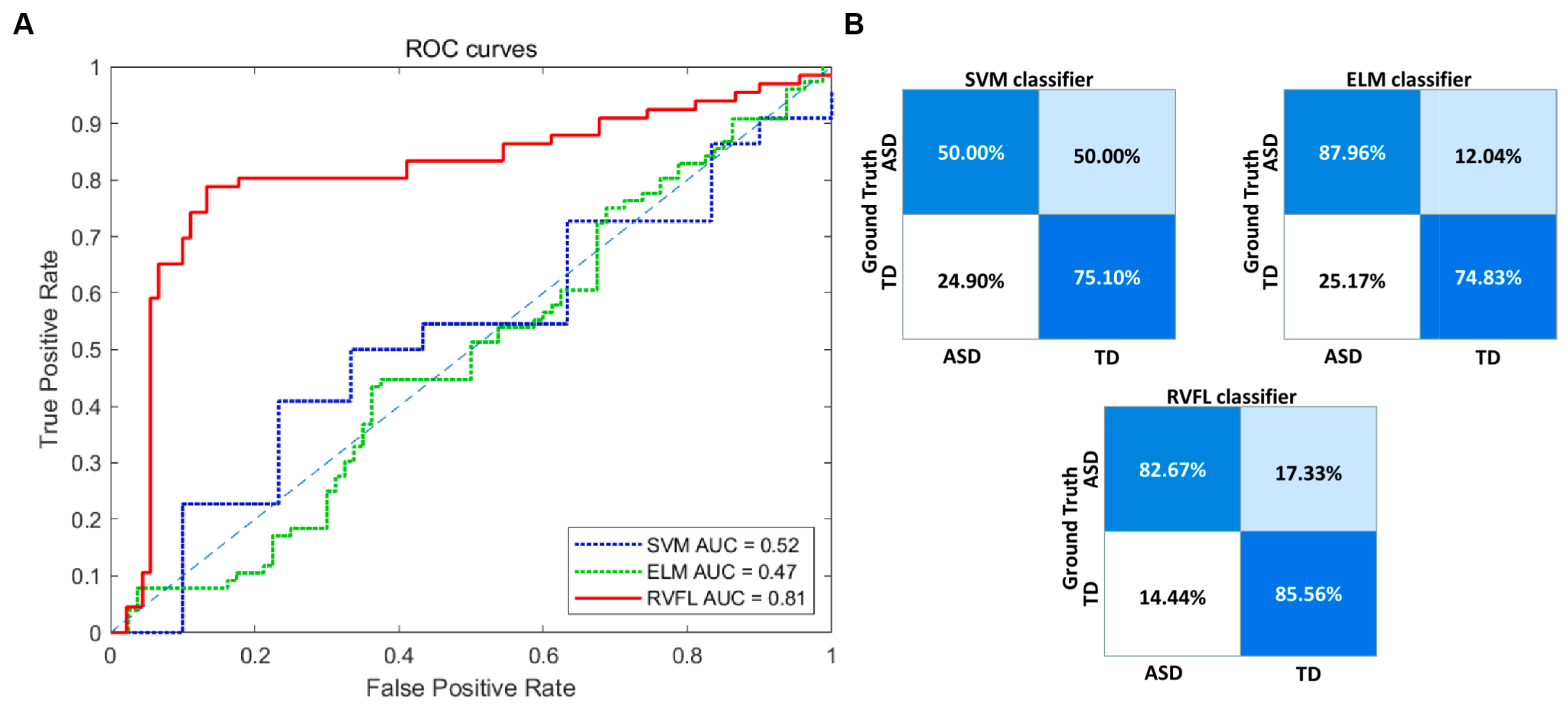
Visualization of the classification performance of different classifiers. **(A)** ROC curve of the three classifiers. **(B)** Confusion matrices of the three classifiers. ROC, receiver operating characteristic; RVFL, random vector functional link; ELM, extreme learning machines; SVM, support vector machine; AUC, area-under-the-curve; ASD, autism spectrum disorder; TD, typically developing.

Figure 3A illustrates the receiver operating characteristic (ROC) plots, and Figure 3B illustrates the confusion matrix using different classifiers. Based on the experimental findings, the RVFL algorithm demonstrated superior classification performance compared to SVM and ELM. It achieved an AUC value of 0.81 in ASD diagnosis. These results indicate that the RVFL algorithm is effective in accurately diagnosing ASD by achieving high accuracy, sensitivity, specificity, and AUC value when RVFL classifier was used.

### 3.2 The performance of the proposed feature in ASD diagnosis

To validate the effectiveness of multimodal brain network features, a comparative analysis was conducted based on the RVFL classifier. As shown in Table 3, the accuracy in ASD diagnosis based on only T1-based or ASL-based network features resulted in worse performance (79.21 and 66.06%, respectively), compared with that using proposed network features together. By using the RVFL classification framework, the ASD diagnosis accuracy was increased prominently. To validate the importance of feature selection, we conducted classification experiments without feature selection and found that accuracy improved by more than 10% after feature selection, demonstrating the significance of feature selection.

**Table 3:** Overall ASD diagnosis performance when different features were utilized.

Features	ACC	SEN	SPC	F1-score	Precision	AUC
T1-based gray matter network	79.21%	71.67%	84.44%	75.98%	77.57%	0.74
ASL-based cerebral blood flow network	66.06%	67.67%	65.56%	61.50%	77.22%	0.65
T1- and ASL-based network	64.79%	50.00%	76.67%	51.37%	66.24%	0.51
Proposed network	84.30%	82.67%	85.56%	81.34%	86.37%	0.81

ACC, accuracy; SEN, sensitivity; SPC, specificity; AUC, area under the curve.

**Figure 4:**
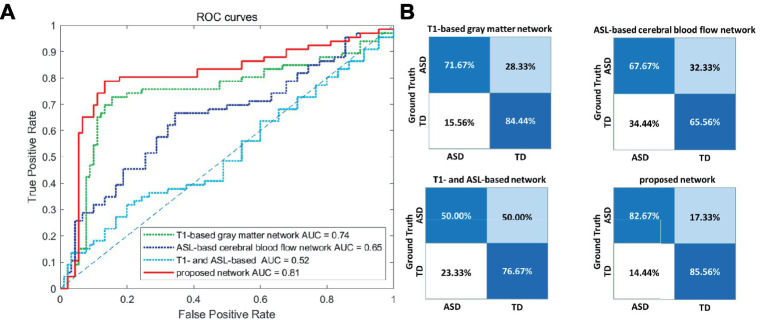
Visualization of the classification performance of different feature types. **(A)** ROC curve of the four feature types. **(B)** Confusion matrices of the four feature types. ROC, receiver operating characteristic; AUC, area-under-the-curve; ASD, autism spectrum disorder; TD, typically developing.

Figures 4A,B illustrate the ROC curve and confusion matrix using different feature types. Based on the experimental findings. In comparison to the other three feature sets, the proposed multimodal brain network fusion features demonstrate a higher AUC value and achieve the best ROC curve performance. Moreover, the confusion matrix illustrates that this feature set enables more accurate identification of individuals with ASD and those without. Furthermore, the study explored the construction of feature sets using unimodal T1, ASL and without feature selection brain network features independently. To summarize, the proposed multimodal brain network fusion features in this study exhibit favorable feature representation and offer clear advantages in distinguishing ASD patients from the neuro-typical population.

### 3.3 The most discriminative brain connectivity features

**Figure 5:**
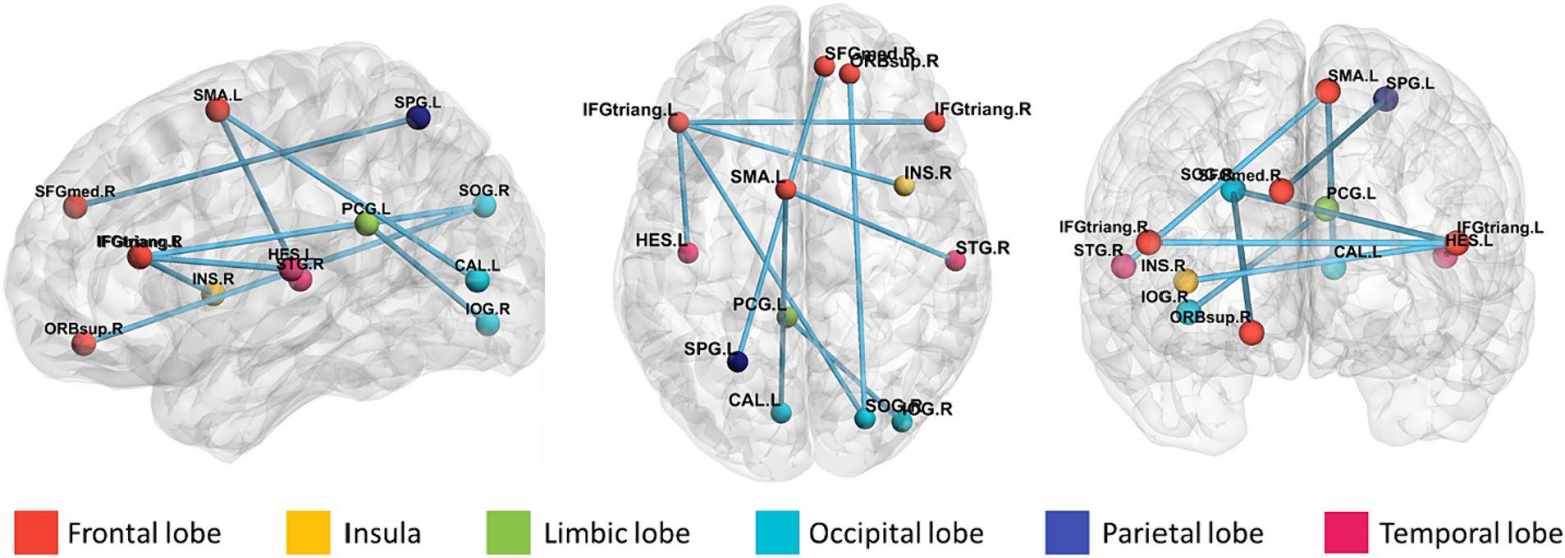
Brain area connectivity map. The top 9 brain area connections closely associated with ASD were found by frequency counts and the corresponding brain area connectivity maps.

**Figure 6:**
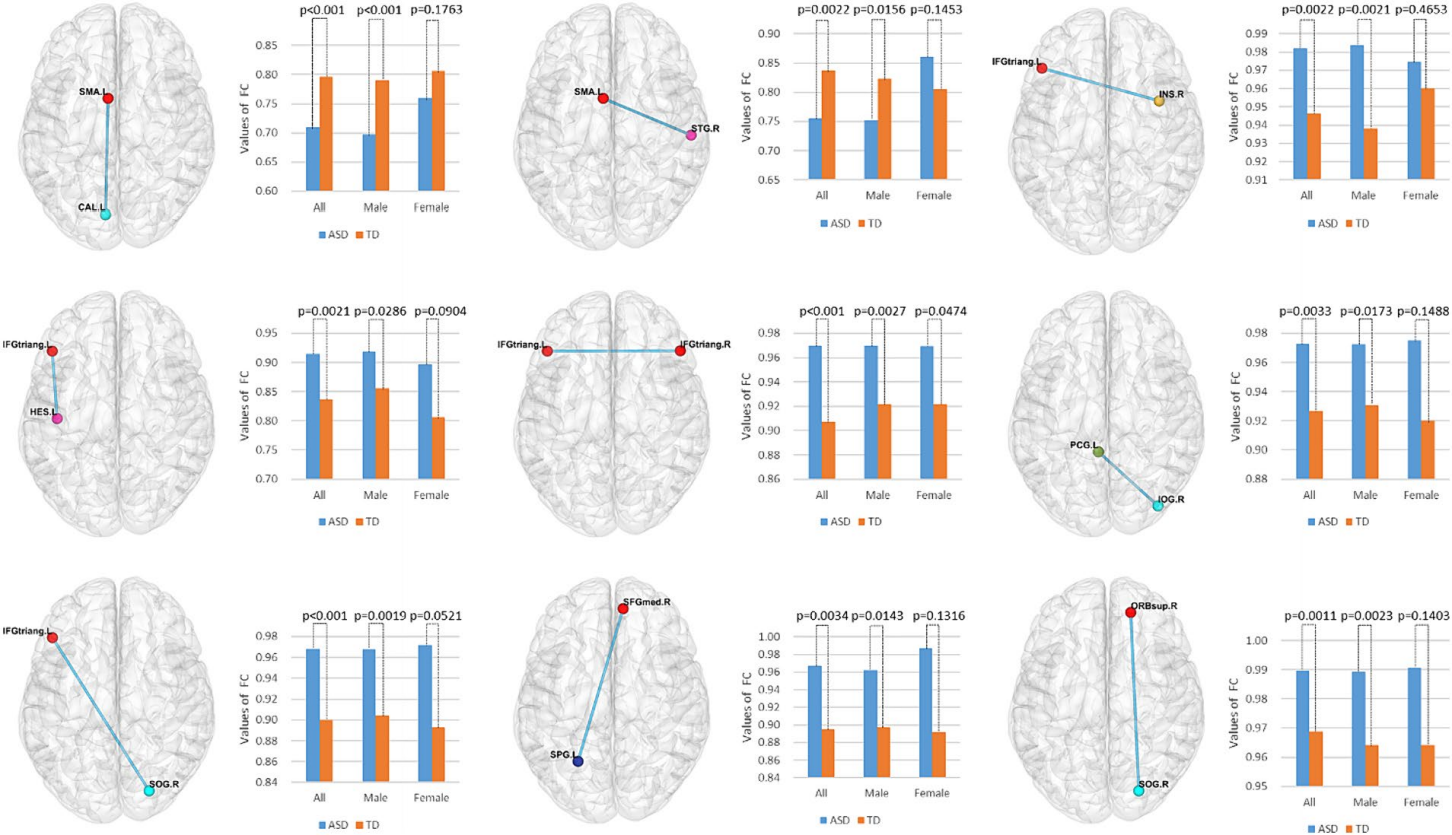
Gender difference analysis of the top nine functional connectivity features. Using a two-sample *t*-test, we evaluated the significant differences among all, male, and female participants for these nine functional connectivity features.

To identify features with significant discriminatory abilities, we employed a five-fold cross-validation approach and performed statistical analyses in each fold. Specifically, we first identified the top 20 brain regions with the smallest *p*-values in each fold. Then, we counted the occurrences of brain region connections in each of the five folds in descending order. Since many brain regions had the same number of occurrences, we compared which brain regions had smaller *p*-values for the same number of occurrences, thus selecting the top 9 brain region FCs with significant differences. As shown in Table 4 and Figure 5, these brain region connections included the following regions: inferior frontal gyrus of the deltoid, supplementary motor area, superior occipital gyrus, inferior occipital gyrus, superior parietal gyrus, transverse temporal gyrus, superior temporal gyrus, supraorbital frontal gyrus, medial superior frontal gyrus, insula, and posterior cingulate gyrus. In this way, we were able to identify more accurately the features of brain regions with significant discriminatory power. To further explore the effect of gender on classification performance, we performed gender difference analysis for the first nine FC features that showed significant differences. As shown in Figure 6, after the validation of the two-sample *t*-test, we found that the *p*-value of only a few FC features is less than 0.01, which indicates that the gender factor has a very limited impact on the classification performance in our study.

**Table 4:** The top 9 discriminative brain connectivity features.

No.	Brain connectivity feature	Brain lobe	Modality
1	L_Supplementary motor area-L_Calcarine cortex	Frontal-limbic	T1
2	L_Supplementary motor area-R_Paracentral lobule	Frontal-frontal	T1
3	L_Inferior frontal gyrus (triangular)-R_Insula	Frontal-insula	T1
4	L_Inferior frontal gyrus (triangular)-L_Precuneus	Frontal-parietal	T1
5	L_Inferior frontal gyrus (triangular)-R_Inferior frontal gyrus (triangular)	Frontal-frontal	T1
6	L_Posterior cingulate gyrus-R_Inferior occipital gyrus	Limbic-occipital	ASL
7	L_Inferior frontal gyrus (triangular)-R_Superior occipital gyrus	Frontal-occipital	T1
8	R_Superior frontal gyrus (medial)-L_Superior parietal gyrus	Frontal-parietal	ASL
9	R_Orbitofrontal cortex (superior)-R_Superior occipital gyrus	Frontal-occipital	T1

L, left hemisphere; R, right hemisphere.

## 4 Discussion

This study proposes an ASD diagnosis method based on T1 and ASL brain network features. The results show that our experiment achieved the best classification performance using RVFL. This is mainly because SVM and ELM encountered challenges in processing highly complex and nonlinear data. SVM relies on finding an effective hyperplane to segment the data, but for ASD brain networks with complex features, finding such a hyperplane can be very difficult (Vapnik, 1964; Huang et al., 2004). Although ELM is suitable for training single-hidden-layer feedforward neural networks, for the complex structure and features of complex brain network data, single-hidden-layer networks may have difficulty capturing them fully. In contrast, the RVFL network structure has demonstrated stronger adaptability in processing such data. By randomly generating hidden layer nodes and weights, combined with regularization techniques, it can more effectively capture the inherent structure of the data, achieving higher classification accuracy.

Our study provides insights into brain network abnormalities in ASD. In this study, we integrated T1- and ASL-based brain network metrics to capture gray matter and cerebral blood flow connectome alteration between pairs of brain regions in ASD. ASL could accurately measure cerebral blood flow in local tissues, providing insights into the mechanisms of ASD and detailed information of distinctions from typical individuals. Multiple brain network features provide comprehensive information of brain structural and functional alteration for ASD. The experiments results show that the combination of multiple brain network features achieves higher classification accuracy compared to using single-modality features only. Therefore, these multiple brain network features based on ASL and T1 MRI techniques display great potential in the early diagnosis of ASD and are expected to become a crucial diagnostic tool in the future.

Our research found that the most significant connectivity features for ASD classification are mainly located in the frontal, occipital, and temporal lobes. The frontal lobe is crucial for children’s social functioning, social cognition, and executive functions, and abnormal CBF values in this region may affect these functions. This is consistent with previous studies, such as those showing ASD patients have social barriers, and a positive correlation between ABC language scores and CBF in the inferior frontal gyrus, indicating potential functional abnormalities or abnormal social tendencies in this area (Wilcox et al., 2002; Ye et al., 2022). However, we also noted inconsistencies with previous research. Some studies have found abnormal CBF values in five brain regions, including the frontal and temporal lobes, which partially aligns with our findings (Mori et al., 2020). But our research also identified functional connectivity abnormalities in the occipital lobe, which was not explored in those studies. These differences may be due to differences in subject age range, sample size, or other research methods. Nevertheless, these findings collectively highlight the tremendous potential of CBF-based brain connectivity studies in exploring biomarkers and diagnostics for ASD.

Previous studies have demonstrated increased cortical thickness in various brain regions in ASD (Hardan et al., 2009; Mak-Fan et al., 2012; Doyle-Thomas et al., 2013). Our findings were in accordance with previous studies, showing that patients with ASD exhibit significant differences in cortical thickness compared to typically developing individuals, particularly in the frontal and occipital cortical regions. Changes in cortical thickness within these language-related areas may reflect language delays or deficits in patients, as well as difficulties in social interaction and autonomic regulation among children with ASD (Khundrakpam et al., 2017; Lucibello et al., 2022). Based on these consistent observations, our study suggests that the method proposed, which relies on brain connectivity features extracted from cortical thickness information, holds promise as a diagnostic tool for identifying neuroimaging biomarkers in ASD.

## 5 Limitations

There are several limitations in this study. First, the limited sample size could affect the generalization of the machine learning model. The need to increase the scanning time for ASL sequences poses challenges, especially for pediatric subjects who are not administered sedation. Second, in this study, the computed cerebral blood flow network matrices are based on Pearson correlation, serving eigenvalues of correlation coefficients as features. Subsequently, graph theory analysis tools can be incorporated to further explore the graph-theoretical properties of the cerebral blood flow network in children with autism spectrum disorder.

## 6 Conclusion

In this study, we performed a preliminary diagnosis of ASD in children based on the T1 gray matter and ASL cerebral blood flow networks, demonstrating the effectiveness of multimodal brain networks in disease recognition. By comprehensively computing different modal brain networks, we successfully extracted comprehensive features for ASD diagnosis and achieved a classification accuracy of 84.30%, which is significantly improved compared to single brain network features. This study not only provides potential neuroimaging biomarkers related to social and executive functions for ASD diagnosis, but also provides new perspectives for our in-depth understanding of the neuropathological mechanisms of ASD. At the same time, this study also provides a useful reference for the application of ASL-based brain networks in prognostic diagnosis. However, we are also aware of some limitations in the study. First, the sample size is relatively small, which may have some impact on the stability and generalization of the results. Future studies can further validate our findings by expanding the sample size. Second, this study mainly focused on the cerebral blood flow network indexes of T1 gray matter and ASL, and future studies can explore the characteristics of more modalities and incorporate more clinical information to improve the accuracy and comprehensiveness of the auxiliary diagnosis.

## Data availability statement

The datasets presented in this article are not readily available because the data are from a private dataset containing data on children in hospital. Requests to access the datasets should be directed to BP, pengbo@sibet.ac.cn.

## Ethics statement

The studies involving humans were approved by Institutional Review Board of the Children’s Hospital affiliated with Jiangnan University. The studies were conducted in accordance with the local legislation and institutional requirements. Written informed consent for participation in this study was provided by the participants’ legal guardians/next of kin.

## Author contributions

ML: Visualization, Writing – original draft, Methodology. WY: Resources, Writing – review & editing. DX: Data curation, Writing – original draft. MW: Investigation, Writing – original draft. BP: Conceptualization, Formal analysis, Methodology, Project administration, Writing – review & editing. HJ: Writing – review & editing. YD: Writing – review & editing.
