# Corrigendum: Diagnosis for autism spectrum disorder children using T1-based gray matter and arterial spin labeling-based cerebral blood flow network metrics

**DOI:** 10.3389/fnins.2024.1480497

**Published:** 2024-09-16

**Authors:** Mingyang Liu, Weibo Yu, Dandan Xu, Miaoyan Wang, Bo Peng, Haoxiang Jiang, Yakang Dai

**Affiliations:** ^1^School of Electrical and Electronic Engineering, Changchun University of Technology, Changchun, China; ^2^Department of Radiology, Affiliated Children's Hospital of Jiangnan University, Wuxi, China; ^3^Suzhou Institute of Biomedical Engineering and Technology, Chinese Academy of Science, Suzhou, China

**Keywords:** autism spectrum disorder, T1-weighted MRI, ASL, gray matter network, cerebral blood flow network, machine learning

In the published article, there was an error in the affiliation for Haoxiang Jiang. Instead of having affiliation 1, “School of Electrical and Electronic Engineering, Changchun University of Technology, Changchun, China,” it should be affiliation 2, “Department of Radiology, Affiliated Children's Hospital of Jiangnan University, Wuxi, China.”

The authors apologize for this error and state that this does not change the scientific conclusions of the article in any way. The original article has been updated.

